# The agronomic mechanism of root lodging resistance and yield stability for sweet corn in response to planting density and nitrogen rates at different planting dates

**DOI:** 10.3389/fpls.2025.1481033

**Published:** 2025-02-11

**Authors:** Qinggan Liang, Hailong Chang, Hongrong Chen, Qingdan Wu, Yuanxia Qin, Zhuqing Wang, Gang Wang, Xuhui Li, Weiwei Chen, Jiantao Wu, Qinnan Wang, Jianqiang Wang

**Affiliations:** ^1^ Hainan Institute of Industrial Technology, Guangdong Academy of Science, Sanya, China; ^2^ Institute of Nanfan and Seed Industry, Guangdong Academy of Sciences, Guangzhou, China; ^3^ School of Breeding and Multiplication (Sanya Institute of Breeding and Multiplication), Hainan University, Sanya, China; ^4^ Key Laboratory for Quality Regulation of Tropical Horticultural Crops of Hainan Province, Hainan University, Haikou, China

**Keywords:** sweet corn, lodging resistance, yield stability, planting density, nitrogen rate, root system architecture

## Abstract

A three-cycle field experiment was conducted to investigate the underlying agronomic mechanism on modulating the root lodging resistance and yield stability of sweet corn in response to the planting density and nitrogen rate during different growth seasons. The experiment comprised two factors with six treatments and was conducted in a split-plot design. Two nitrogen (N) rates (200 kg ha^-1^, N200; 150 kg ha^-1^,N150) applied to the main plots and three planting densities (20 cm plant space, D20; 25 cm plant space, D25; and 30 cm plant space, D30; 60 cm rows space for all plots) as subplots. The results indicated that the plants in N150D25 presented better root system architecture, greater root biomass, and more roots per plant. These effects are mediated by the underlying metabolism of endogenous phytohormones, which balance the absorbing and anchoring function of the root system. This further improved the development of plant crown architecture, including stem nodes and ear leaf traits, and further coordinated dry matter dynamics and lignin metabolism between the root and shoot organs. These observations may account for the resistance of the roots to lodging in this treatment. The maximum yield output was achieved in the plants under N150D25 via a significant increase in individual ear fresh weight, kernel number per row, and grain number per ear via path analysis. Compared with that of N200D30 (local field management), the yield of N150D25 plants increased by 22.33%–30.00% during the three growing seasons. Notably, the yield stability was achieved by significantly reducing the coefficient of variation (CV) of cob length and diameter, ear diameter, kernel row number per plant and grain number per plant. Among these factors, the planting date had a considerable effect on ear fresh weight, cob fresh weight, ear length, cob diameter, cob length and kernel row number by significantly increasing the degree of variation. This finding indicated that the planting date is a crucial factor that should be accounted in field crop management. Our findings provide a scientific basis for high-yield production of sweet corn in tropical regions during the “off season” period.

## 1 Introduction

Under continued global climate change, extreme climate disasters such as windstorms, waterlogging events, and heat stress occur frequently, which present severe threats to global food security (Gitz et al., 2016). Both stem lodging and root lodging, which are common challenges facing cereal production and cause yield or even harvest losses (Mahoney et al., 2015), are caused by heavy rainstorms that occur during the summer, especially in cereals dominating tropical or subtropical regions (Niu et al., 2016). Hence, harnessing the lodging resistance potential of crops via genetic breeding and cropping techniques is urgently needed. Stem lodging occurs when stalks break at or below the ear-bearing node of the stem and strongly interferes with the transport of water and nutrients between the root and above-ground parts (Shah et al., 2021). Complex structure carbohydrates such as cellulose, hemicellulose, and lignin, the main components of the stem, significantly affect lodging resistance (Li et al., 2023). In addition, stem lodging is strongly influenced by plant height, ear height, the ear position coefficient, and the diameter/length ratio of stem nodes (Zhang et al., 2021). However, regarding root lodging, which is defined as the inclination of plants above 30°from the vertical direction, frequently occurs during the grain-filling stage (Xue et al., 2020). Root morphological parameters, such as root number, brace root number, total root length, root diameter, and root angle, are factors that significantly influence root lodging (Zheng et al., 2023a). Plant hormones metabolism contribute greatly to the regulation of various aspects of root system architecture (Li et al., 2022). Previous studies have revealed that hormones such as indole-3-acetic acid (IAA), trans-zeatin-riboside (ZR), and jasmonic acid (JA) considerably promote the initiation and elongation of primary and lateral roots (Wen et al., 2023; Wang et al., 2023; Zheng et al., 2023b). In contrast, the gibberellic acid (GA) and abscisic acid (ABA) may act as suppressors of root formation (Gaspar and Coumans, 1987; Zeng et al., 2021). Notably, stem lodging and root lodging commonly occur together (Erndwein et al., 2020). Hence, coordinating the interaction between the roots and above-ground parts is crucial to improve the lodging resistance of crops (Niu et al., 2022).

To meet the food demand for the growing global population, increasing the crop’s planting density has been proven to be an effective cropping method to increase the grain yield per unit area of the field (Hemathilake and Gunathilake, 2022). This method, however, has the disadvantage of increasing the risk of lodging to crops (Shah et al., 2021). High-density planting significantly increases the stalk lodging rate by reducing the lignin and cellulose contents in the stem, simultaneously, the root system development is limited (Li et al., 2021). These phenomena can be associated with a fierce competition between individuals or groups of plants for source capture, which resulting in decreased accumulation and distribution of dry matter for the stems and roots development (Zhang et al., 2020). However, low planting density for crop development will cause sunlight loss and futher reduce light-use efficiency, which leading to yield losses (Jafari et al., 2024). An optimal planting density, conversely, can shape a reasonable canopy structure for efficient light distribution to increase the light-use efficiency for plants at both the group and individual levels. Hence, improve crop yield by coordinating the interaction between the individual and group of plants and the development of the root and above-ground parts (Liao et al., 2022).

Nitrogen (N) is a macro-nutrient that plays an essential role in plant growth and development. However, its excessive use has caused environmental pollution and soil degradation, which are common phenomena that occur widely across the globe (Tyagi et al., 2022). Therefore, nitrogen management is crucial for promoting the growth and development of field crops. The optimal combination of planting density and nitrogen application can not only alleviate the pressure of plant competition for nutrient and sunlight capture (Haque and Sakimin, 2022) but also improve plant lodging resistance via efficient dry matter accumulation and allocation to vegetative and reproductive organs (Li et al., 2022).

The planting date arrangement, being an important factor, which is responsible for yield improvement. The selection of an optimum planting date would maximize crop yields because, on the one hand, it can overcome the yield penalty caused by the uncertainties in climatic factors (Yang et al., 2020). On the other hand, the variable hybrid seeding has its own cumulative temperature, which is a factor influencing sprouts and the kernel set (Dziwulska-Hunek et al., 2020). However, off-season crop production is an effective way to increase farmer income (de Souza Nóia and Sentelhas, 2020). Thus, the extension of the planting windows for crops by adjusting planting techniques is highly important and deserves to be investigated.

Sweet corn, a special fresh-use corn, is widely cultivated throughout the world and satisfies the demands of the large population for its nutritional value (Subaedah et al., 2021). The ears of sweet corn, which has a shorter growth period than other cereals do, can be harvested after about 80–85 days of transplanting. Hence, sweet corn is a popular crop that has great potential for cultivation in the non-harvest season, e.g., hot summers, in Hainan Province. However, owing to the frequent occurrence of windstorms, lodging stress has become a severe threat to sweet corn cultivation. Therefore, improving lodging resistance is a key factor that maintains yield output and enhances yield stability. However, related studies are limited, and the agronomic mechanisms underlying lodging resistance under nitrogen and planting density management practices, however, are not well understood. The aim of this study was to examine the effects of planting density and nitrogen rate on sweet corn root system architecture, plant crown structure, dry matter dynamics, endogenous phytohormone levels, and lignin metabolism in root-shoot organs, along with the roles of these factors in root lodging resistance as well as yield and yield stability of sweet corn.

## 2 Materials and methods

### 2.1 Experimental site

Three-cycle field experiments were conducted at the Breeding Base of the Institute of Nanfan & Seed Industry, Guangdong Academy of Science, Yazhou District, Sanya, Hainan Province, China (18°21′30″N and 109°9′54″E). The study region has a tropical marine monsoon climate, and the climate data of the three growing seasons are provided in **Supplementary Figure S1**. The soil type of the field is light loam (1.25 g/cm^3^; 20.7% silt content), and its physical and chemical properties in the 0–30 cm tillage layer are shown in **Supplementary Table S1**.

### 2.2 Experimental design

Three sets of field experiments were carried out during the period of 2023–2024, with the first round (2023-1) starting on March 5, 2023, and finishing on May 20, 2023, while the second round (2023-2) was conducted on May 8, 2023 and completed on July 23, 2023, and the third round (2023-3) commenced on December 25, 2023, followed by harvesting performed on March 20, 2024. A two-factor split-plot design was adopted, and the plots were arranged as a randomized complete block design (RCBD) with three replications. Two nitrogen levels were set in the main plot, including 200 kg ha^–1^ (N200; CK) and 150 kg ha^–1^ (N150; 25% reduction in CK), while the subplots consisted of three planting densities, including 83,375 plants ha^–1^, 66,700 plants ha^–1^, and 55,583 plants ha^–1^, with spacings of 20 cm (D20), 25 cm (D25), and 30 cm (D30; CK), respectively, and were coupled with a row spacing of 60 cm. The two sets of treatments were as follows: N200D20, N200D25, and N200D30 (CK) and N150D20, N150D25, and N150D30. The fertilizers used included 75 kg ha^–1^ pure P and 100 kg ha^–1^ K was applied as the basal fertilizer at the sowing stage. The fertilizer resources used were urea containing 46% N (SINOPEC, Co., Ltd.), calcium superphosphate containing 16% P_2_O_5_ (SDIC Xinjiang Lop Nur Potassium Salt Co., Ltd.), and potassium sulfate with 52% K_2_O (Guangdong Zhanhua Group Co., Ltd.). Field management practices for pest and weed control, as well as irrigation methods, were implemented according to local practices.

### 2.3 Plant materials

The sweet corn cultivar “JBT15” was use in this experiment, which purchased from Qingdao Jinmama Agricultural Technology Co., Ltd., Qingdao, Shandong Province, China. Abiotic stress-tolerant corn has been widely cultivated in tropical and subtropical regions in China. The normal planting density of this variety reach to 45,000–60,000 plants. ha^–1^ in field soils with a medium fertility level in tropical regions. The seeds were sown in 105-well plugs containing nutrient-rich substrates, and at the “three-leaf” stage, the seedlings were transplanted to the experimental field.

### 2.4 Sampling methods

Two sampling times were performed at the jointing stage (8-leaf period) and tasseling stage (15-leaf period). Nine representative plants were selected from each treatment for measuring the SPAD values and leaf nitrogen contents via a TYS-4N hand-held chlorophyll meter (Beijing Jinkelida Electronic Technology Co., Ltd.). Next, five representative plants were selected from each treatment and subjected to the following procedures. The root system of individual plants distributed in a soil pot (20 cm wide × 30 cm long × 30 cm deep) was obtained and then washed with running tap water to remove any present silt, immersed in distilled water, and dried. Thereafter, the dried root samples from each treatment were mixed thoroughly and collected in centrifuge tubes, followed by immediate immersion in liquid nitrogen for 30 min and storage in an ultra-low temperature freezer at –80°C for the subsequent hormone assay.

### 2.5 Measurement procedures

#### 2.5.1 Agronomic traits

During both the jointing and tasseling stages, the root system of three plants from each treatment was obtained, and their morphological parameters, such as root length, number of root tips, root volume, root surface area, root diameter, and root projected area, were measured using a root system scanner (EPSON EXPRESSION 10000XL, China). Moreover, the fresh weight and number of both crown roots and brace roots were determined. Furthermore, the specific root length and root length density were calculated according to the following formulas:


Specific root length =total root length /root dry biomass

(Kawai et al., 2022).


Root length density =total root length / unit of soil volume

(Osborne et al., 2020).

During the filling stage, three representative plants were selected from each treatment. The above-ground plant tissues were separated, and the plant height, ear height, coefficient of ear height, diameter of the 3^rd^ stem base node, ear leaf length, and ear leaf width were measured with a measuring tape or a vernier scale. Moreover, to measure the ear leaf angle, a protractor was used, and the leaf area, ear position coefficient, flatness of the 3^rd^ stem node, and cross-sectional area of the 3^rd^ stem node were calculated via the following formulas:


Leafarea=leaf length×leaf width×0.75

(the empirical coefficient) (Li et al., 2021).


Ear position coefficient=ear height / plant height

(Zhao et al., 2021).


$$The\;flatness\;of\;the\;3^{rd}\;stem\;node=a/b$$

(Dong et al., 2024).


$$Cross-sectional\;area\;of\;the\;3^{rd}\;stemnode=\pi \times a^{3}\times b/4$$

(Wu et al., 2020).

where “a” and “b” indicate the outer diameters of the minor and major axes in an oval cross section, respectively.

The fresh root system and above-ground parts of each plant were harvested. All the samples were oven-dried at 105°C for 30 min to kill the microorganisms and then at 80°C to obtain a constant dry weight. The dry matter allocation to the above-ground organs and the root/shoot ratio were calculated as follows:


Dry matter allocatio=dry weight of different organs / whole plant dry weight×100%

Root / shoot ratio=root dry matter / shoot dry matter

#### 2.5.2 Lodging rate

On May 8, 2023 (2023–1; the filling stage), June 8, 2023 (2023–2; the 8-leaf stage), rainstorms occurred in the field, which induced root lodging, whereas no stem lodging was observed in the whole field. The root lodging rate was calculated using the following formula.


Lodging rate=the number of lodging plants per plot/total number of plants per plot×100%

Root lodging is classified into two levels, namely, Grade 1 (G1; the plant leans more than 30–60°) and Grade 2 (G2; the plant leans more than 60–90°), on the basis of the degree of root lodging severity (Niu et al., 2016).

#### 2.5.3 Yield and yield components

In our experiments, the three middle rows of each treatment plot were selected for yield determination. Twenty-one days after pollination for each treatment was arranged for harvesting. The first round of the experiment was conducted on May 20, 2023, whereas the second round started on July 10, 2023, and the third round commenced on March 20, 2024. The ears were harvested and weighed, and then, the number of ears per plot and the average fresh weight of the ears were calculated. Seven representative ears of plants from each plot were subsequently selected to investigate commercial characteristics, including the corn ear length, ear diameter, cob length, cob diameter, row number per ear, grain number per row, grain number, and length of the bare tip. In addition, the coefficient of variation of these parameters was calculated as follows:


$$Coefficient\;of\;variation\left(CV\right)=standard\;deviation\left(SD\right)\;/\;mean\times 100\%$$

(Xu et al., 2021).

The smaller CV is, the better the consistency of the data.

#### 2.5.4 Endogenous phytohormone concentrations

The endogenous hormone concentrations in the roots were determined via enzyme-linked immunosorbent assay (ELISA) via monoclonal antibodies. In accordance with the methods of Si et al. (2022), the contents of endogenous phytohormones were determined via extraction from fresh roots (1.0 g), which were then powdered in liquid nitrogen. The mouse monoclonal antigens and antibodies against IAA, ZR, ABA, GA_3_, and JA-me used in the ELISA were provided by the Phytohormone Research Institute, China Agricultural University.

#### 2.5.5 Determination of lignin contents

The lignin contents in the stems and roots were determined via a lignin assay kit (Suzhou Comin Biotechnology Co., Ltd., Suzhou, China). In accordance with Liang et al. (2023), 15 mg of the sample from three replications of each treatment was transferred to a 10-mL test tube with a glass stopper containing 1000 µL of reagent 1 mixed with 40 µL of perchloric acid, and then, each tube was placed in a water tank at 80°C for 40 min. The tubes were shaken multiple times at 10-min intervals. Thereafter, 1000 µL of reagent 2 was added to each tube and mixed well, and the mixture was centrifuged at 5000 rpm for 2 min. The resulting supernatant (40 µL) was collected in a fresh test tube, and 1960 µL of reagent 3 was added. Three tubes with quartz sand used in the test samples were considered the control. All the tubes containing the mixture were exposed to 280 nm ultraviolet light. Finally, the lignin content was calculated using the following formula:


$$Lignin\;content\;\left(mg/gdryweight\right)=\left(\text{Δ}A-0.0068\right)÷0.0694\times Vt\times 10-3÷W\times T$$

where ΔA=Asample−Actrl ; Vt = total volume of the reaction system; W = sample weight; and T = dilution ratio.

### 2.6 Statistical analysis

Three-way analysis of variance (ANOVA) was used to determine significant differences among the treatments at the significance levels of P < 0.05, P < 0.01 and P < 0.001. Path analysis was used to reveal the relationships between yield and yield components and between root traits and the root lodging rate. The analysis of the collected data was performed via SPSS software (version 19), and figures were generated via GraphPad Prism software version 8.4.2 (for Windows).

## 3 Results

### 3.1 Endogenous hormone contents in roots and role in root architecture

**Figure 1:**
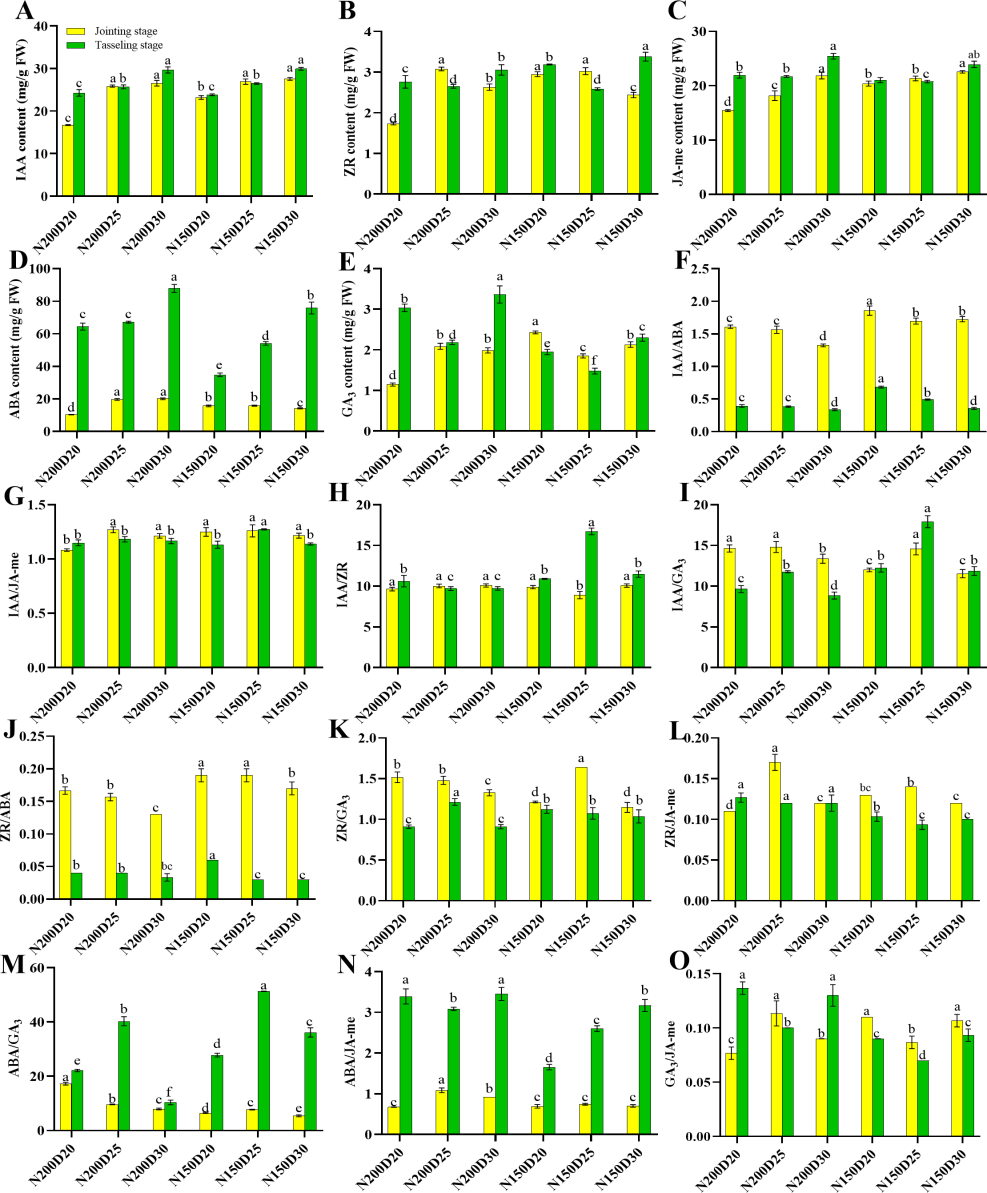
Effects of different planting densities and nitrogen rates on phytohormone contents in the root during both the jointing and tasseling stages. **(A)**, IAA; **(B)**, ZR; **(C)**, JA-me; **(D)**, ABA; **(E)**, GA_3_; **(F)**, IAA/ABA; **(G)**, IAA/JA-me; **(H)**, IAA/ZR; **(I)**, IAA/GA_3_; **(J)**, ZR/ABA; **(K)**, ZR/GA_3_; **(L)**, ZR/JA-me; **(M)**, ABA/GA_3_; **(N)**, ABA/JA-me; **(O)**, GA_3_/JA-me. Data are presented as means ± SE. Statistically significant differences among treatments evaluated by the Duncan multiple range test at P < 0.05 are indicated by different letters.

**Figure 2:**
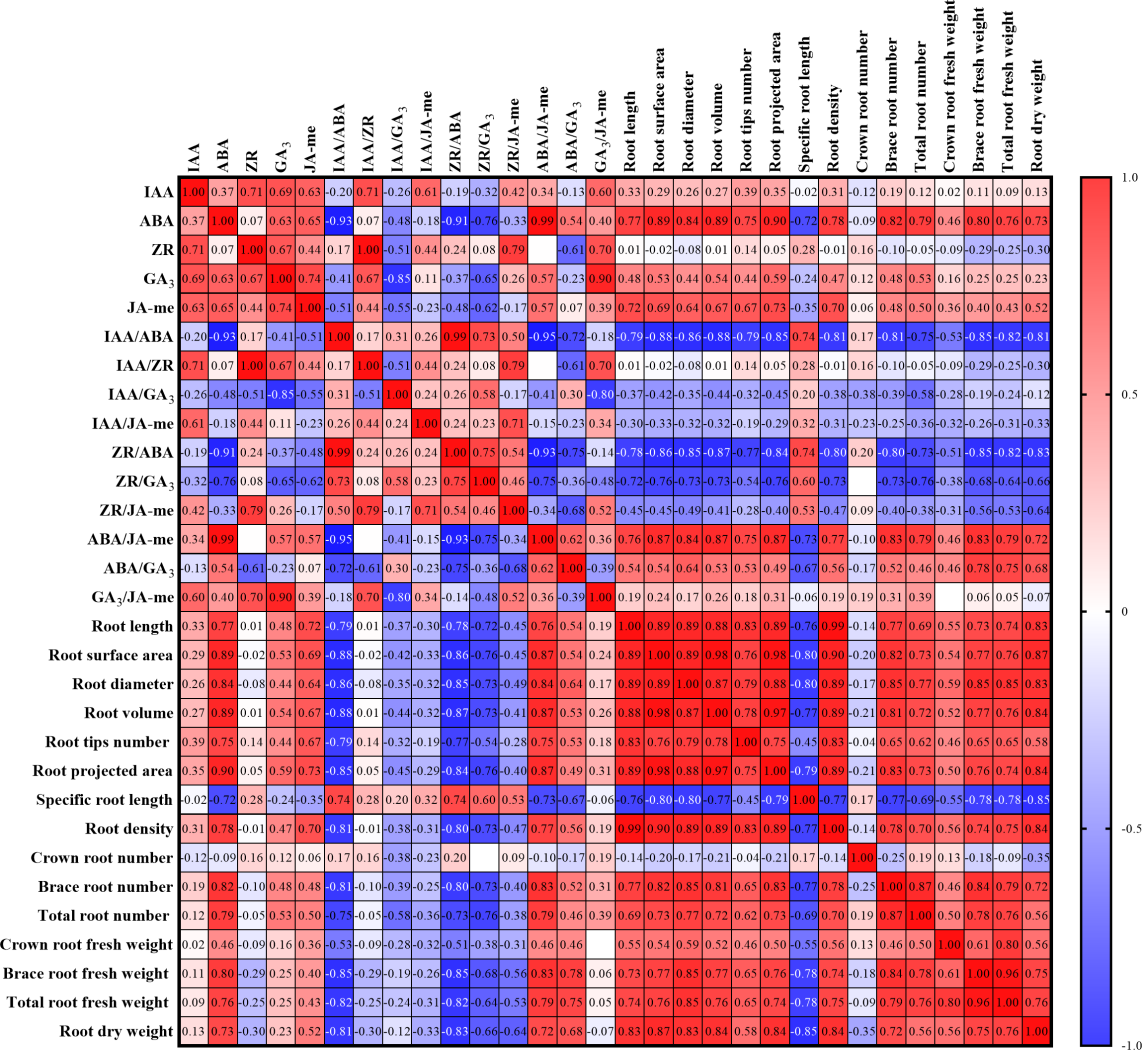
Pearson correction analysis between the root system architecture and root biomass characteristics and contents of endogenous phytohormones in roots at different planting densities and nitrogen application rates; Red cells indicate the positive correlation between the two sets of parameters, while blue cells denote the negative correlation. The number in each cell represents the R^2^ value.

The planting density and nitrogen rate had comparable effects on endogenous phytohormone metabolism in the roots (**Figure 1**). First, the contents of IAA, JA-me, and ABA in the roots significantly increased with decreasing of planting density at both growth stages (P < 0.05; **Figure 1**). Compared with those in the plants from the other treatments, the ZR and GA_3_ contents in the plants from the D25 treatment were significantly greater during the jointing stage but significantly lower at the tasseling stage (P < 0.05). Gradual decreases in the IAA/ABA and ZR/ABA ratios were observed with decreasing planting density. Compared with those in the other treatments, significant increases in IAA/JA-me, IAA/ZR, IAA/GA_3_, ZR/GA_3_, ZR/JA-me, and ABA/GA_3_ ratios were recorded in D25-treated plants (P < 0.05), whereas the GA_3_/JA-me ratio exhibited the opposite trend. The ABA/JA-me ratios in all the treatments were significantly greater during the tasseling stage than during the jointing stage (P < 0.05). Correlation analysis between root architecture, root biomass, and metabolism of endogenous phytohormones were showed in **Figure 2**. The root system architecture traits, except for specific root length and root biomass and number, were negatively correlated with IAA/ABA, IAA/ZR, IAA/GA_3_, IAA/JA-me, ZR/ABA, ZR/GA_3_, and ZR/JA-me but positively correlated with ABA/JA-me, ABA/GA_3_, and GA_3_/JA-me (P < 0.05; **Figure 2**). Moreover, the root system architecture characteristics, with the exception of specific root length and root traits, except for crown root number, were positively correlated with the contents of IAA, ABA, GA_3_, and JA-me in the roots but negatively correlated with the ZR content (P < 0.05).

### 3.2 Root system architecture

**Figure 3:**
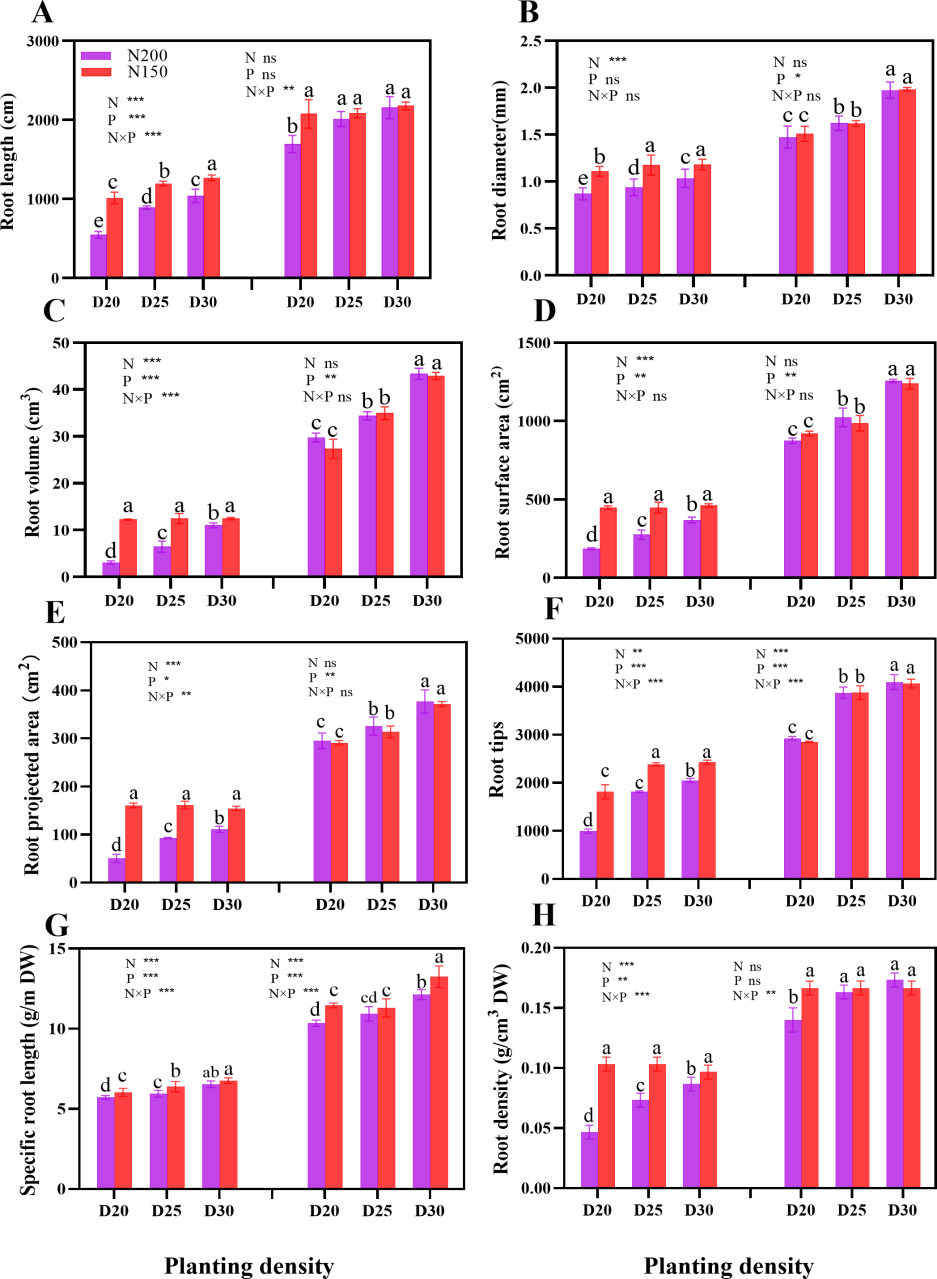
Effects of different planting densities and nitrogen rates on root system architecture during both the jointing and tasseling stages. **(A)**, Root length; **(B)**, root diameter; **(C)**, root volume; **(D)**, root surface area; **(E)**, root projected area; **(F)**, root tips; **(G)**, specific root length; **(H)**, root density. Data are presented as means ± SE. Different letters show significant differences among treatments determined according to the Duncan test (P < 0 .05). Significant difference levels are presented by *P < 0.05; **P < 0.01, ***P < 0.001, ^ns^ no significance

During the jointing stage, the root length, root diameter, root volume, root surface area, root projection area, number of root tips, specific root length, and root density of sweet corn significantly increased with decreasing planting density at the same nitrogen application level. Notably, the plants under the N150 treatment presented significantly greater average values of these parameters than the N200-treated plants (P < 0.05; **Figure 3**). However, in terms of specific root length, different nitrogen application rates did not significantly differ at a certain planting density during the tasseling stage (P > 0.05; **Figure 3**).

### 3.3 Root biomass

**Figure 4:**
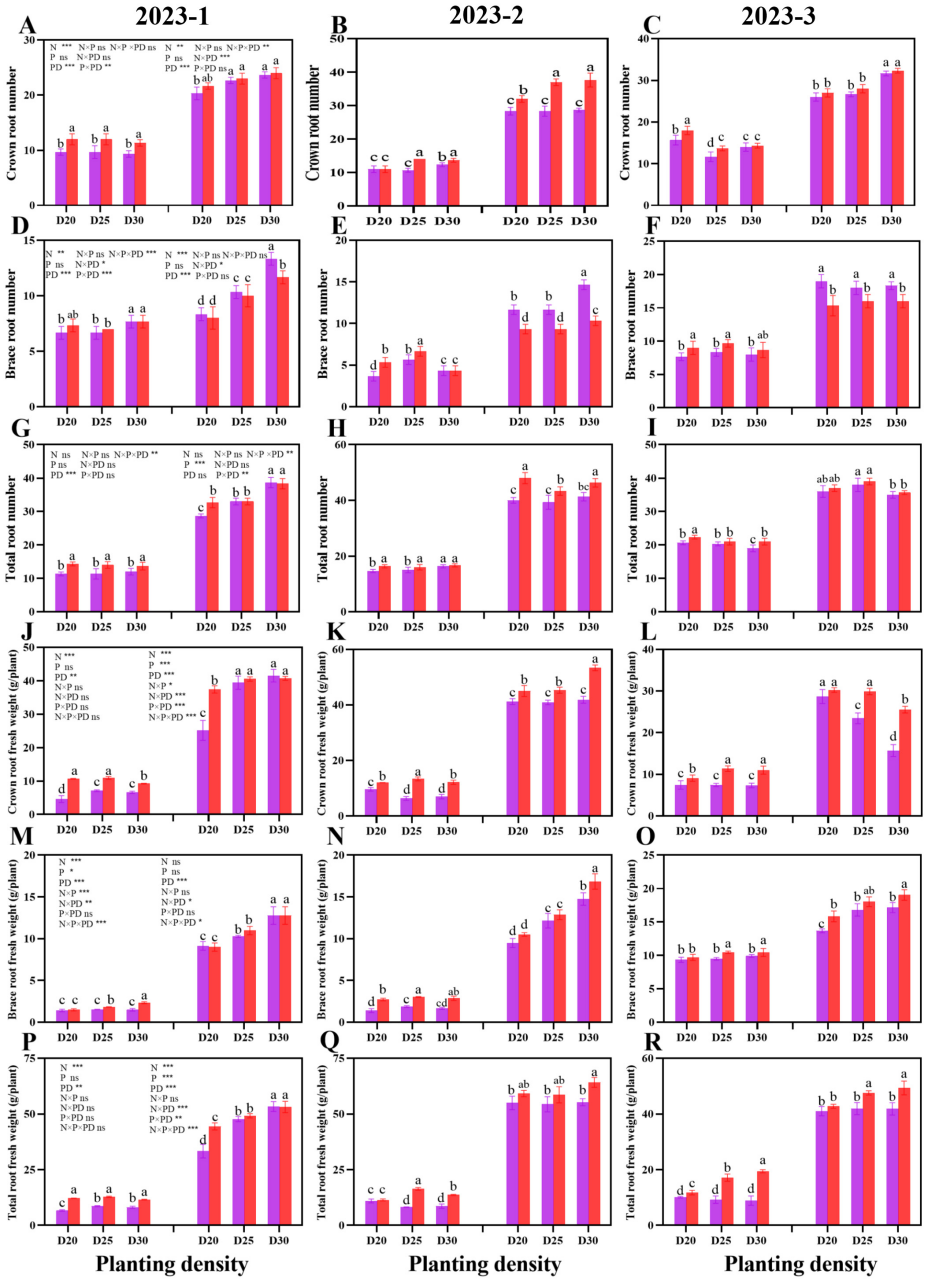
Effects of different planting densities and nitrogen application rates on root biomass during both the jointing and tasseling stages. **(A–C)**, crown root number; **(D–F)**, brace root number; **(G–I)** , total root number; **(J–L)**, crown root fresh weight; **(M–O)**, brace root fresh weight; **(P–R)**, total root fresh weight. Data are presented as means ± SE. Statistically significant differences among the treatments determined based on the Duncan test (P < 0 .05) are marked by different letters.Significant difference levels are presented by *P < 0.05; **P < 0.01, ***P < 0.001, ^ns^ no significance.

A similar trend was observed for the root biomass of sweet corn in the three rounds of the field experiment (**Figure 4**). During both growth stages, the crown root number, brace root number, and total root number significantly increased as the planting density decreased at the same nitrogen rate (P < 0.05; **Figure 4**). During the jointing stage, the N150 treatment resulted in significantly greater values of the abovementioned parameters than did the N200 treatment (P < 0.05). However, during the tasseling stage, the plants in N200 presented significantly greater brace root numbers than did the N150 plants at the same population density (P < 0.05). The crown root number and total root number of the plants in N150, however, were greatest. Similarly, at a given planting density, significantly greater values of root fresh weight were recorded for N150 plants than for N200 plants (P < 0.05; **Figure 4**).

### 3.4 Plant height and development of the 3^rd^ internode

**Figure 5:**
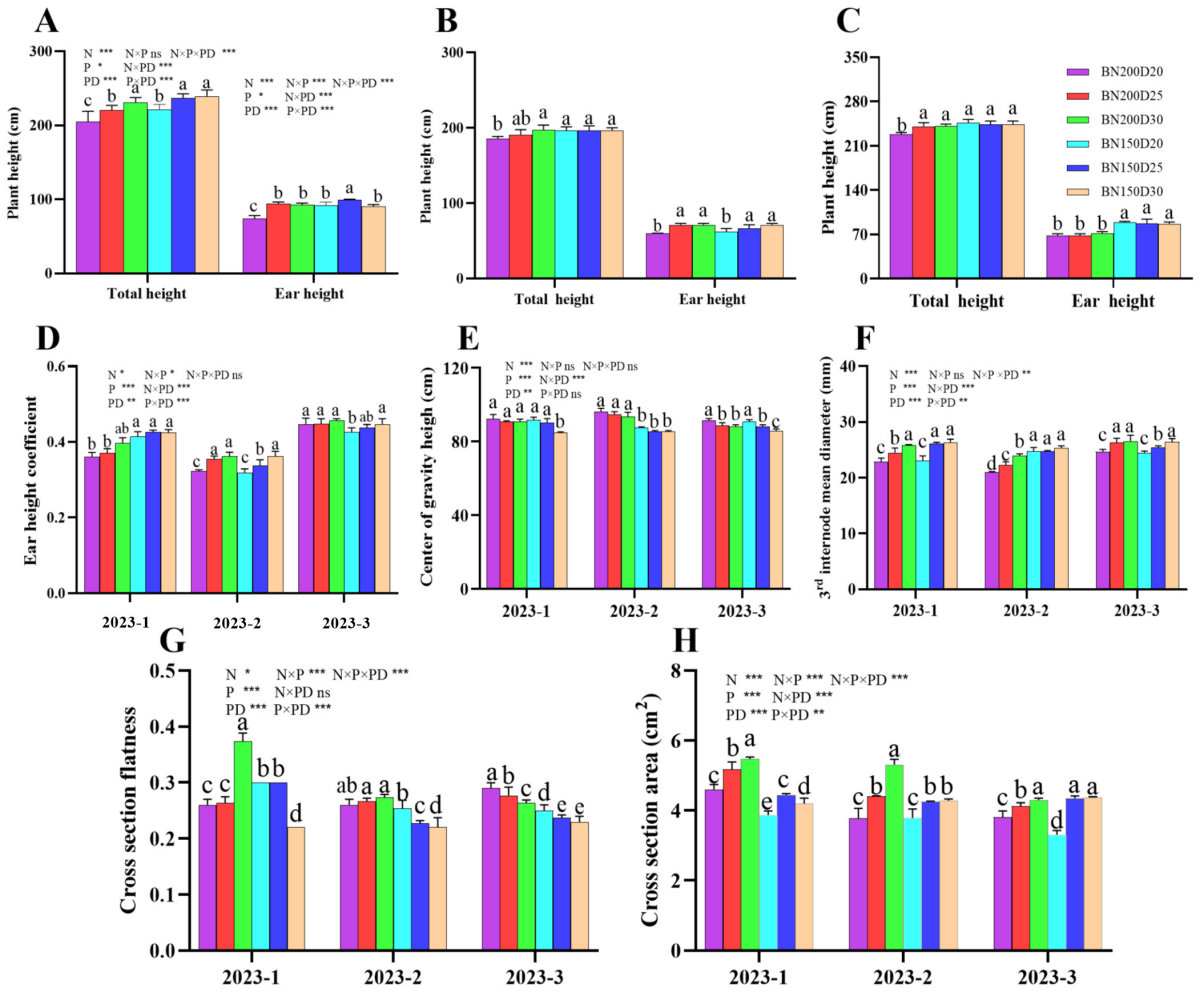
Effects of various planting densities and nitrogen application levels on plant height and stem development. **(A–C)**, plant height; **(D)**, ear height coefficient; **(E)**, center of gravity height; **(F)**, 3^rd^ internode mean diameter; **(G)**, cross section flatness; **(H)**, cross section area. All data are presented as means ± SE. Different letters denote significant differences among treatments compared according to the Duncan test (P < 0 .05). Significant difference levels are presented by *P < 0.05; **P < 0.01, ***P < 0.001, ^ns^ no significance.

Both the planting density and the nitrogen rate significantly affected the plant height and development of the stem node of sweet corn (**Figure 5**). The plant height, ear height, coefficient of ear height, stem diameter of the 3^rd^ internode, and cross-sectional area of the 3^rd^ internode significantly increased with decreasing planting density when the same nitrogen level was applied (P < 0.05; **Figure 5**). However, the N150 treatment had the opposite effect on the flatness of the cross-sectional area of the 3^rd^ stem internode and the height of the center of gravity of the plants (P < 0.05; **Figure 5**).

### 3.5 Ear leaf traits

**Figure 6:**
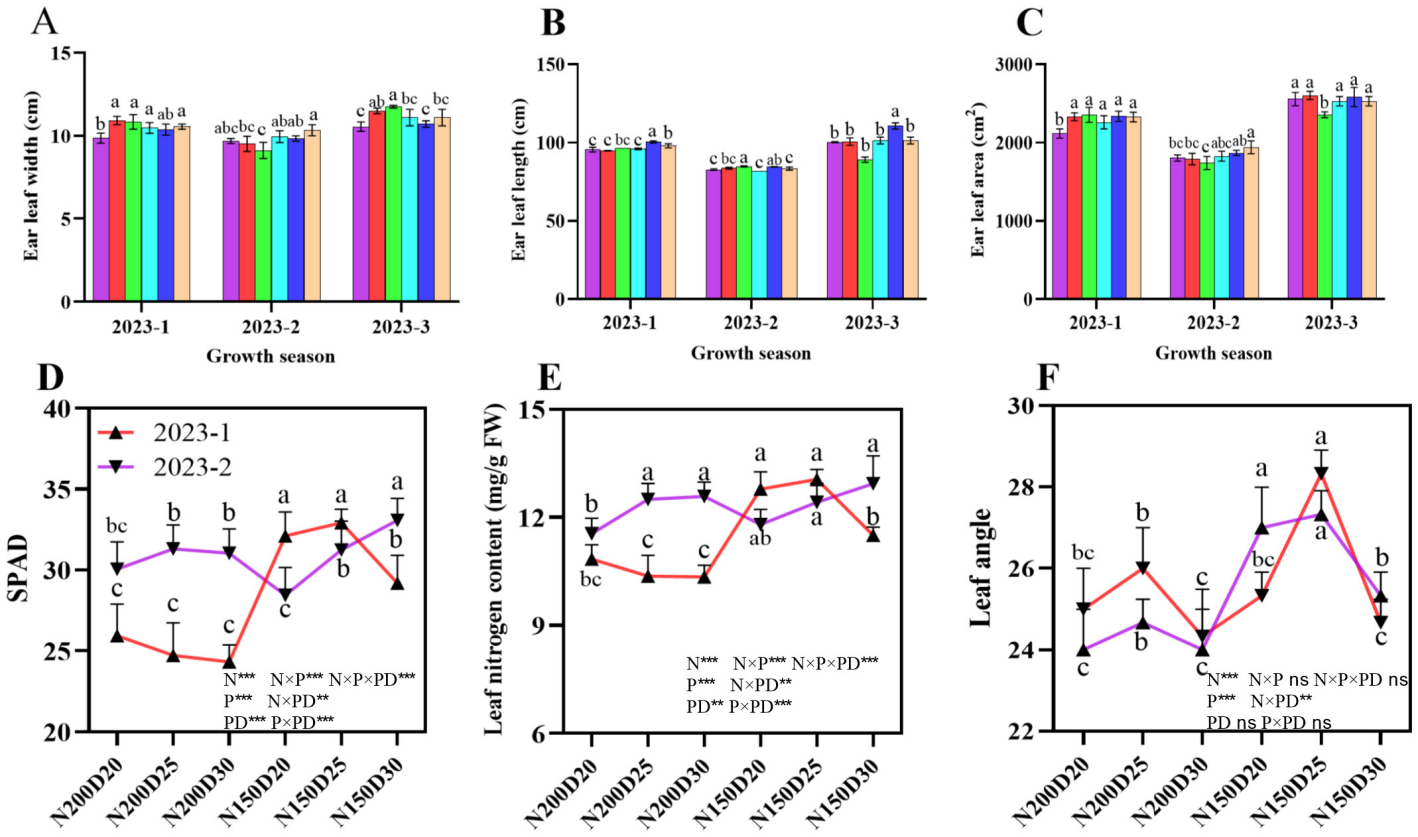
Effects of different planting densities and nitrogen application rates on the photosynthetic traits of the ear leaf. **(A)**, ear leaf width; **(B)**, ear leaf length; **(C)**, ear leaf area; **(D)**, ear SPAD value; **(E)**, ear leaf nitrogen content; **(F)**, ear leaf angle. Data are presented as means ± SE. Significant differences among the treatments determined by the Duncan test at P < 0 .05 are shown by different letters. Significant difference levels are presented by *P < 0.05; **P < 0.01, ***P < 0.001, ^ns^ no significance.

Ear leaf development followed a similar trend across all three sets of field experiments (**Figure 6**). The leaf width, leaf length, and leaf area at the ear position significantly increased with decreasing planting density at the same nitrogen addition level (P < 0.05; **Figure 6**). Notably, the greatest ear length was achieved in the N150D25 treatment, which was significantly different from the results of the other treatments (P < 0.05; **Figure 6**). In addition, the SPAD value and nitrogen content in the ear leaf, as well as the ear leaf angle, significantly increased in N150 plants compared with their N200 counterparts (P < 0.05; **Figure 6**), with the maximum value observed in N150D25 plants.

### 3.6 Dry matter dynamics

**Figure 7:**
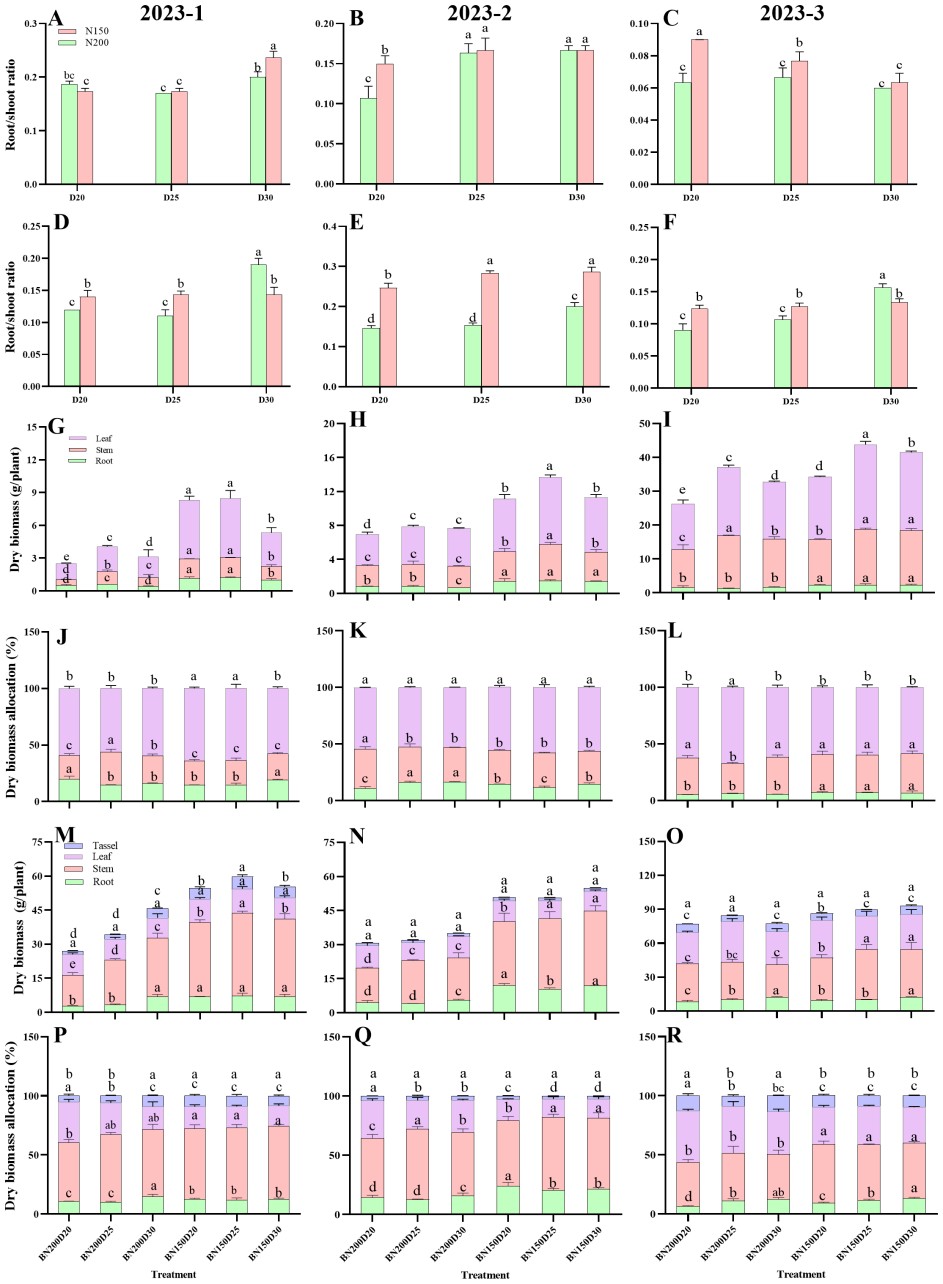
Effects of planting densities and nitrogen rates on dry matter dynamics of plants during the jointing and tasseling stage. **(A–C)**, root/shoot ratio in jointing stage; **(D–F)** root/shoot ratio in tasseling stage; **(G–I)**, dry biomass allocation in jointing stage; **(J–L)**, dry biomass in tasseling stage; **(M–O)**, dry biomass in tasseling stage; **(P–R)**, dry biomass allocation in tasseling stage. Data are presented as means ± SE. Different letters indicate significant differences among the treatments evaluated by the Duncan test at P < 0 .05.

The dynamics of the dry matter showed a similar trend over the three sets of the field experiment (**Figure 7**). In both growth stages, significantly greater values of the root-shoot ratio, root dry biomass, stem dry biomass, leaf dry biomass, and root-shoot dry biomass ratio of the plants in the N150 treatment were noted for the N200 plants when the same planting density was applied (P < 0.05; **Figure 7**). Compared with N150 plants, N200 plants presented significantly greater dry biomass allocation to leaves during both growth stages (P < 0.05), and the biomass of the dried tassel did not significantly differ among the treatments in the 2023-2 and 2023-3 field trials (P > 0.05).

### 3.7 Lignin contents in the stem and root

**Figure 8:**
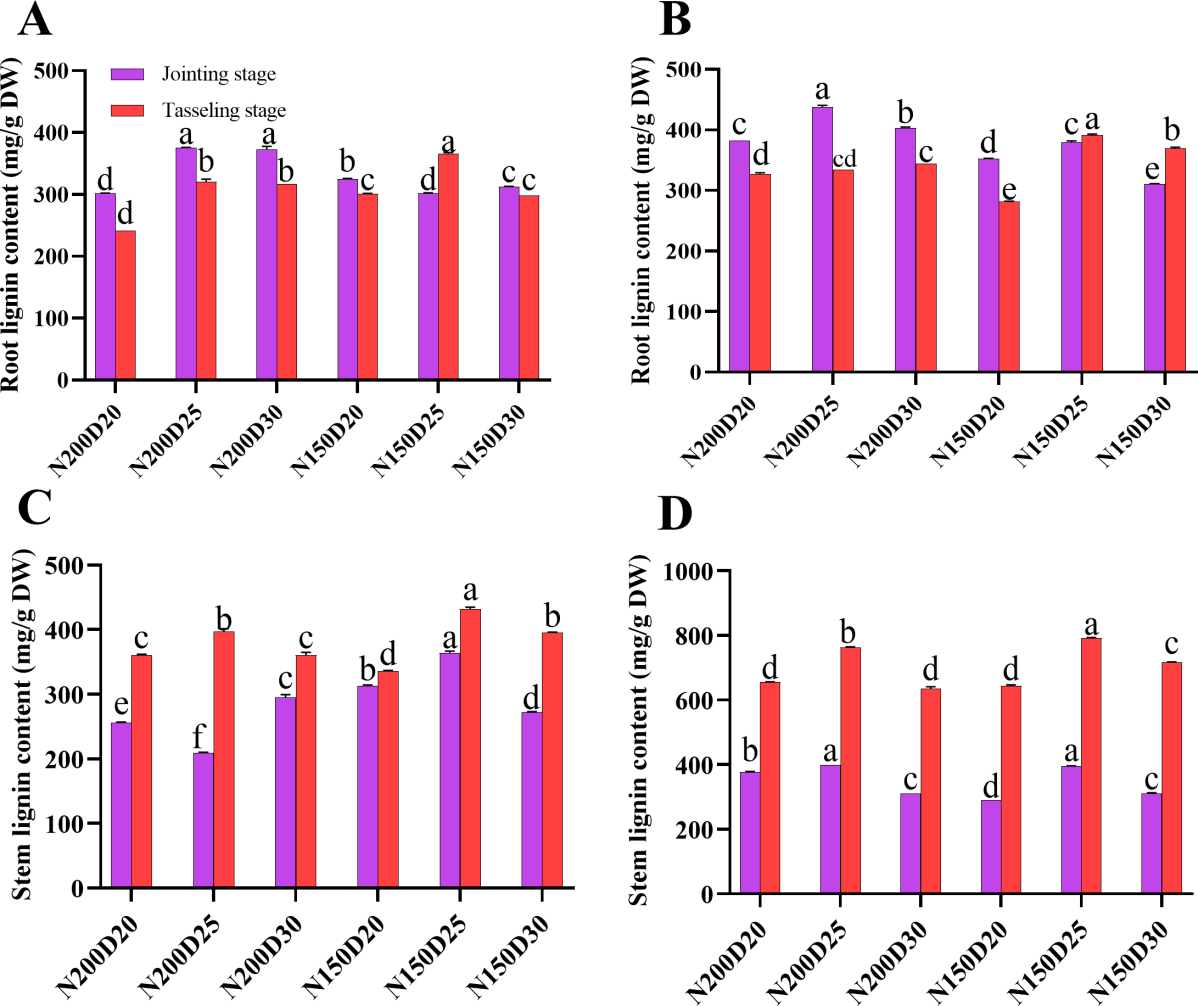
Effects of different planting densities and nitrogen application levels on lignin biosynthesis in plant tissues. **(A, B)**, root lignin content; **(C, D)**, stem lignin content. Data are presented as means ± SE. Statistically significant differences among treatments determined by the Duncan multiple range test at P < 0 .05 are indicated by different letters.

In the two rounds of the field experiment, the lignin contents of the roots and the stem nodes showed similar trends (**Figure 8**). During the jointing stage, N200D25 plants presented a significantly greater lignin content in their roots than did those from the other treatments, whereas during the tasseling stage, significantly greater values were detected in N150D25 plants (P < 0.05; **Figure 8**). Furthermore, the lignin content in the stem nodes increased with increasing growth period, and the lignin content in the stems was the highest in the N150D25 treatment (P < 0.05).

### 3.8 Lodging rate

**Figure 9:**
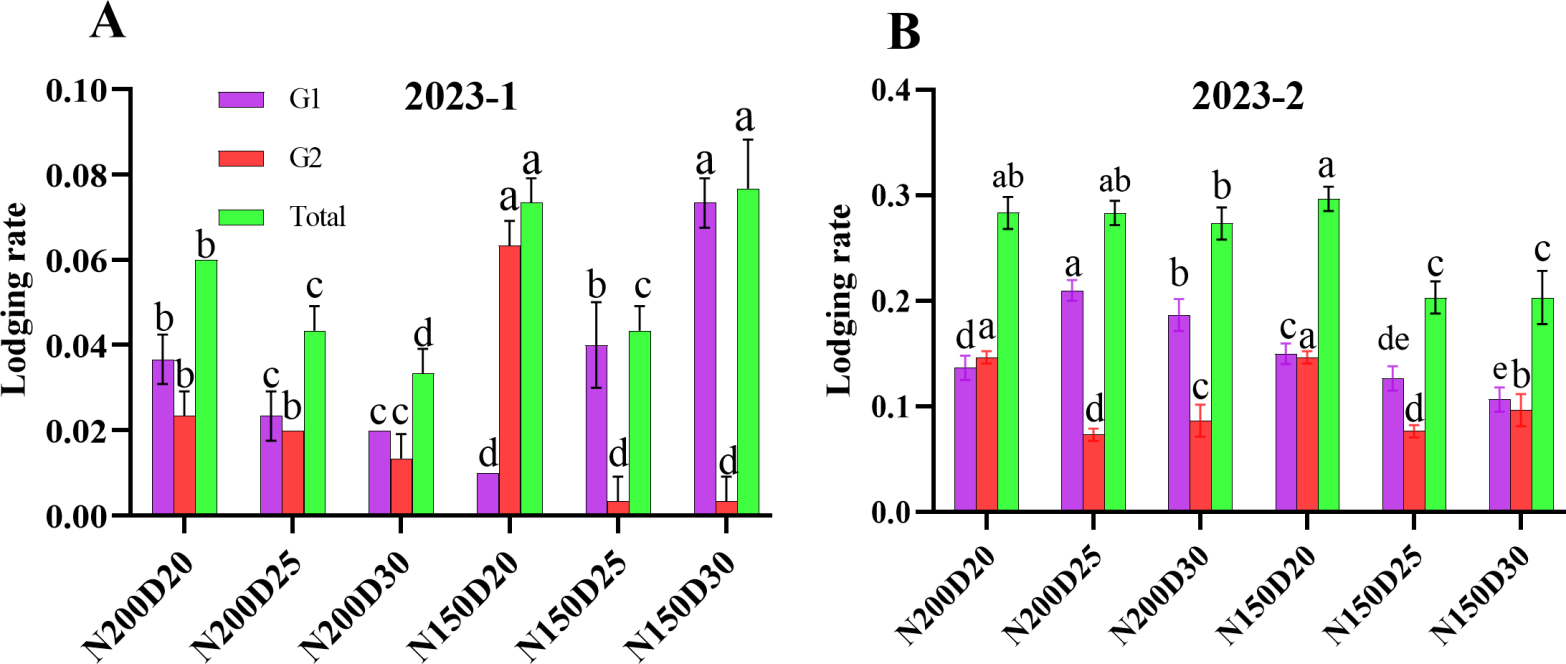
Effects of different planting densities and nitrogen addition rates on the root lodging rate **(A)** 2023-1 at tasseling stage; **(B)** 2023-2 at jointing stage. Data are expressed as means ± SE. Significant differences among the treatments evaluated by the Duncan test (P < 0.05) are indicated by different letters.

No lodging occurred in the 2023-3 field experiment. In both 2023-1 and 2023-2 rounds of the field experiment, the total root lodging rate was lowest in the plants under the N150D25 treatment. At the same nitrogen rate, the plants at D20 presented a significantly greater root lodging rate than did those at the other planting densities because of relatively greater G1 and G2 level root lodging (P < 0.05; **Figure 9**). In each treatment, the G1-level root lodging rate was lower than the G2-level root lodging rate.

### 3.9 The characteristics of commercial ears

**Figure 10:**
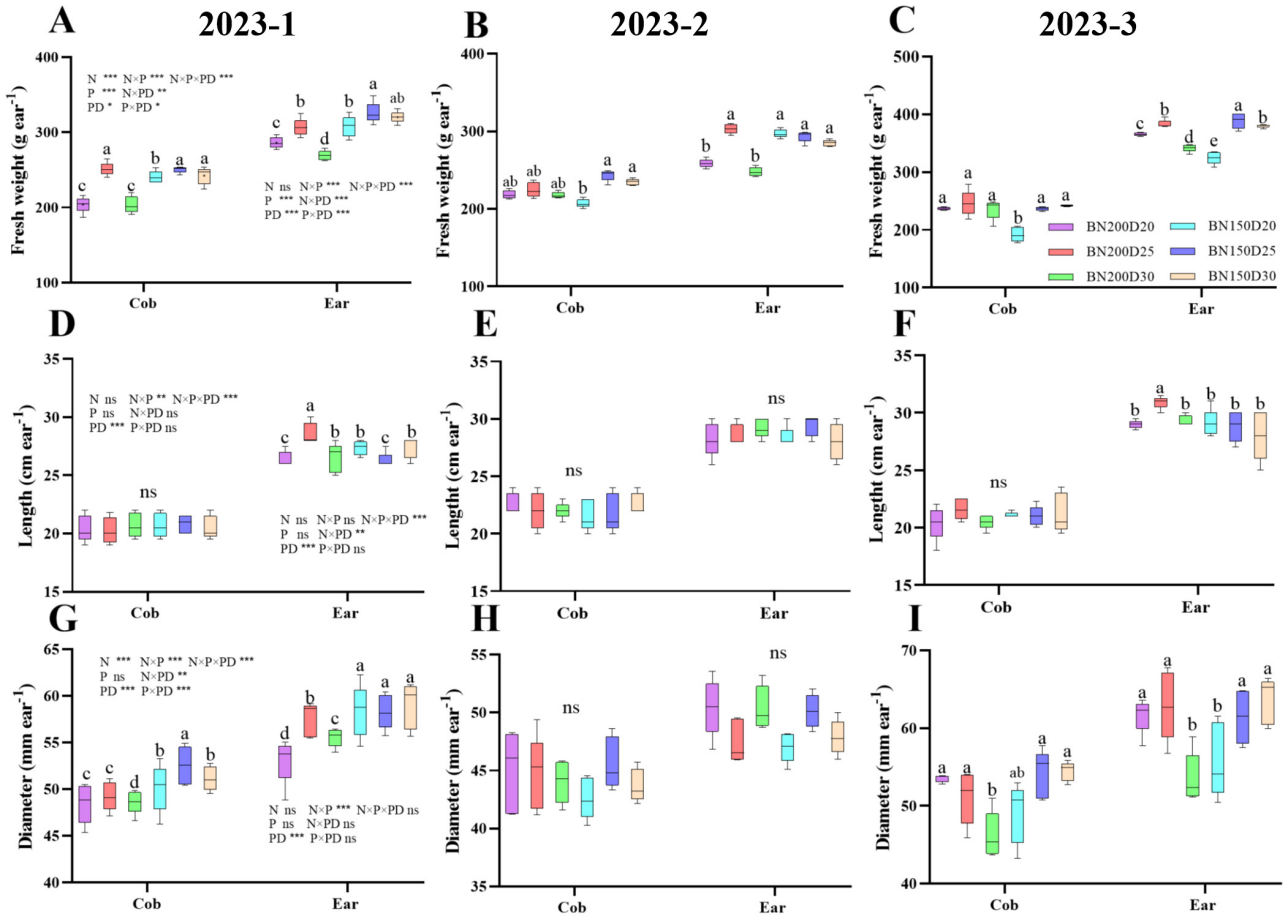
Effects of various planting densities and nitrogen application levels on the characteristics of commercial ears. **(A–C)**, ear fresh weight; **(D–F)**, ear length; **(G–I)**, ear diameter. Data are expressed as means ± SE. Different letters indicate significant differences among the treatments determined by the Duncan test (P < 0 .05). Significant difference levels are presented by *P < 0.05; **P < 0.01, ***P < 0.001, ^ns^ no significance.

The characteristics of commercial maize ears are closely associated with their final yield. Throughout the growing season, significant increases in the fresh weight and diameter of single cobs and single ears of plants were observed under the D25 treatment at the same nitrogen application level (P < 0.05; **Figure 10**). Moreover, the cob length did not differ among the treatments, and the ear length was greatest in the N200D25-treated plants, which was significantly different from the results of the other treatments (P < 0.05).

### 3.10 Yield and yield components

**Figure 11:**
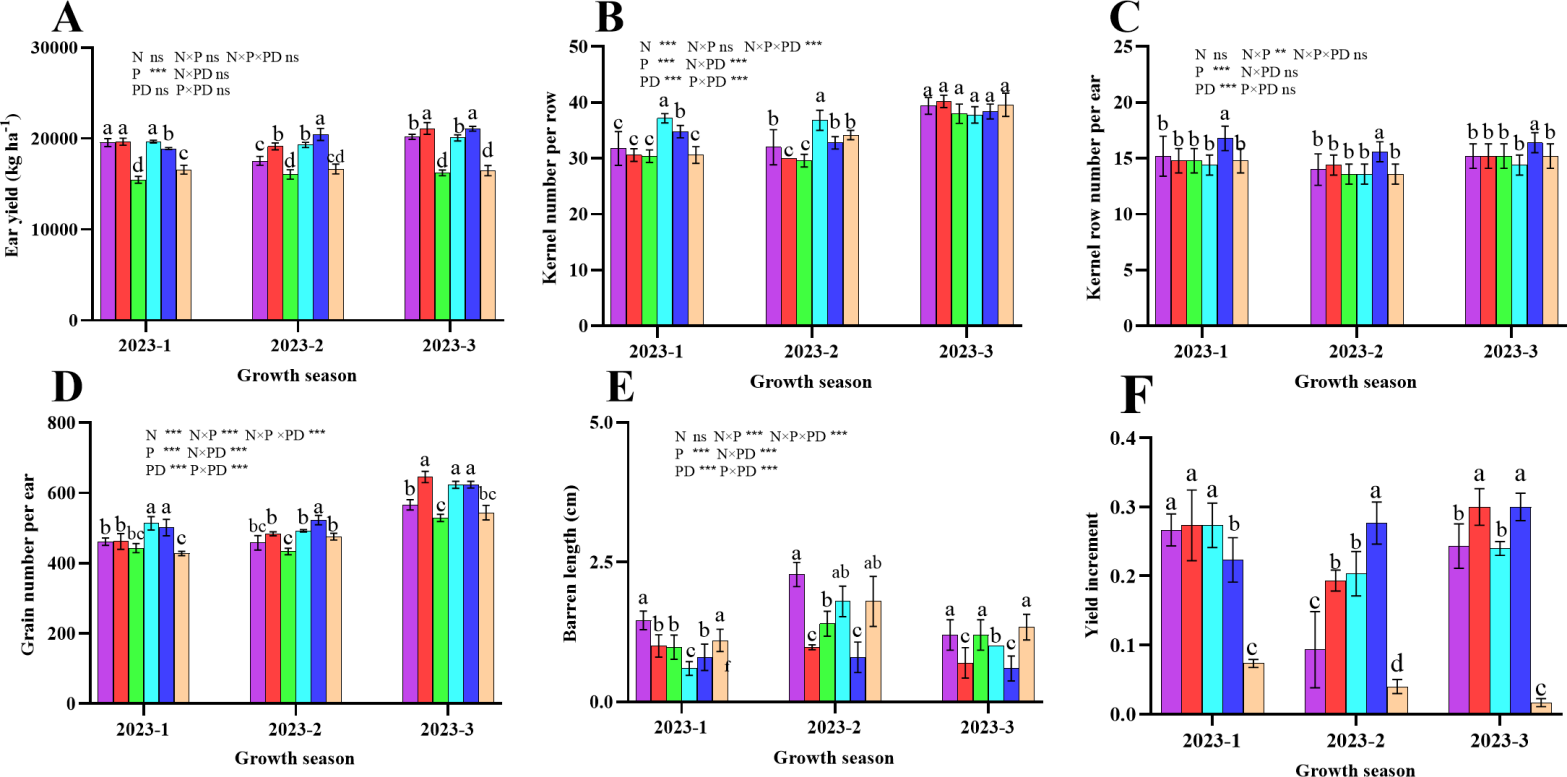
Effects of various planting densities and nitrogen application levels on ear yield and yield components. **(A)**, ear yield; **(B)**, kernel number per row; **(C)**, kernel row number per ear; **(D)**, grain number per ear; **(E)**, barren length; **(F)**, yield increment. Data are expressed as means ± SE. Significant differences among treatments, detected by the Duncan test (P < 0 .05), are presented by different letters. Significant difference levels are presented by *P < 0.05; **P < 0.01, ***P < 0.001, ^ns^ no significance.

**Figure 11** shows that the three rounds of the field experiment had similar effects on the yield and yield components of sweet corn. The fresh ear yield of sweet corn, the number of grains per ear, and the number of rows per ear were significantly greater in the plants from the D25 treatment (P < 0.05; **Figure 11**). However, with decreasing planting density, the kernel number per row significantly decreased in the 2023-1 and 2023–2 field trials (P < 0.05), whereas in 2023-3, the various treatments presented no significant difference (P > 0.05). Notably, the ear barren tip length was significantly lower in D25 plants than in plants from other planting density levels (P < 0.05). Compared with those of the plants in the control N200D30 treatment, the yield increases in the N200D20, N200D25, N150D20, N150D25, and N150D30 treatments were 26.67%, 27.33%, 27.33%, 22.33%, and 7.33%, respectively, at 2023-1, whereas at 2023-2, percent yield increases of 9.33%, 19.33%, 20.33%, 27.67%, and 4.00%, respectively, were observed, and at 2023-3, the percent increases in yield were 24.33%, 30.00%, 24.00%, 30.00%, and 1.67%, respectively. Furthermore, the nitrogen rates contributing to corn yield did not differ between the growing seasons of 2023-1 and 2023-3, but in 2023–2, the yield was significantly greater in N150 than in N200 (P < 0.05). Furthermore, the D25 treatment significantly differed from the other planting densities in terms of yield (P < 0.05).

### 3.11 Ear traits and yield stability

Ear characteristic uniformity is crucial for stable yield production. N150 plants presented lower average coefficients of variation (CVs) for ear fresh weight, cob fresh weight, ear length, cob length, ear diameter, cob diameter, kernel row number per ear, kernel number per row, grain number, and barren tip length. The CV values for cob length, ear diameter, cob diameter, kernel row number per ear, kernel number per row and grain number per ear were lowest for plants from the N150D25 treatment, indicating that uniformity was significant (**Table 1**). The ANOVA results indicate that the planting density, nitrogen rate and planting date and their interactive effects significantly affected these parameters, with the exception of the number of grains per ear (**Table 1**). Among the impact factors, planting date had the greatest influence on the CV of ear fresh weight, cob fresh weight, ear length, ear diameter, cob length, cob diameter, and kernel row number per ear by increasing the proportion of variation that contributed to these mentioned ear traits (**Table 1**).

**Table 1:** Effects of different planting densities and nitrogen rates on the coefficient of variability (CV) of yield component uniformity (%).

PD	N	P	EFW	CFW	EL	ED	CL	CD	KRN	KNPR	GN	BTL
2023-1	N200	**D20**	2.24 c	11.19 a	2.26 c	4.59 b	4.34 a	3.51 a	10.33 b	8.94 b	6.54 d	12.47 a
		D25	3.42 a	3.70 d	4.32 a	4.43 b	3.92 b	2.50 b	6.38 d	8.75 b	2.64 e	11.10 b
		D30	2.72 b	9.77 b	4.42 a	7.97 a	4.59 a	2.70 b	10.87 b	10.07 a	19.66 a	9.12 c
	N150	D20	2.85 b	8.91 c	2.32 c	4.61 b	3.41bc	2.49 b	13.41 a	7.90 c	7.78 c	7.62 d
		D25	3.71 a	9.73 b	2.51 c	3.64 c	2.95 cd	2.51 b	9.71 c	4.66 d	1.29 f	4.81 e
		D30	3.90 a	10.52 ab	2.99 b	4.39 b	2.45 d	3.40 a	10.34 b	7.82 c	8.56 b	9.60 c
2023-2	N200	D20	1.89 f	2.41 c	6.84 c	8.87 a	4.71 b	6.92 b	9.31 a	8.89 c	4.12 b	9.68 d
		D25	3.71 d	2.27 c	6.85 c	3.40 c	4.68 b	5.81 c	5.61 b	2.74 d	1.68 c	10.76 c
		D30	7.99 c	9.80 b	5.57 d	4.74 d	5.24 a	7.26 a	6.00 b	10.75 a	7.66 a	13.69 a
	N150	D20	2.29 c	9.95 b	10.52 a	7.82 b	2.59 c	3.80 e	5.55 b	8.79 c	4.54 b	9.53 d
		D25	9.94 a	9.86 b	7.61 b	4.12 d	2.45 c	2.42 f	5.84 b	2.84 d	1.51 c	12.68 b
		D30	8.96 b	11.23 a	2.46 d	6.92 c	2.51 c	4.35 d	5.87 b	10.75 a	4.37 b	13.55 a
2023-3	N200	D20	12.53 a	11.35 d	7.89 a	6.72 c	6.42 b	7.40 a	6.95 c	11.60 a	6.72 b	8.70 c
		D25	12.34 a	16.29 b	1.54 d	5.41 d	3.65 e	6.02 c	6.74 c	5.06 d	2.83 d	10.62 b
		D30	7.89 d	9.98 c	6.44 b	6.64 c	5.16 c	6.04 c	10.87 b	11.82 a	5.67 c	11.11 b
	N150	D20	10.88 c	19.87 a	7.80 a	11.11a	7.63 a	6.80 b	13.55 a	11.65 a	7.82 a	4.38 d
		D25	11.78 b	12.38 c	2.90 c	3.96 e	4.88 d	2.99 e	6.82 c	8.10 c	2.14 d	4.47 d
		D30	12.64 a	11.86 cd	6.61 b	4.38 d	7.71 a	4.85 d	6.60 c	9.78 b	6.59 b	13.57 a
Proportion of Variation (%)												
PD			71.18	23.93	34.13	28.85	22.83	26.73	34.87	11.22	13.63	19.09
N			3.01	2.68	0.44	8.54	13.66	6.59	0.72	3.87	4.03	13.08
P			6.01	9.07	9.68	4.99	7.33	2.11	7.22	6.70	40.89	22.71
PD*N			0.82	25.02	1.60	26.23	3.64	14.76	6.85	7.30	4.17	5.73
PD*P			10.53	9.82	41.20	5.66	19.56	4.33	6.66	20.89	16.37	15.50
N*P			2.06	4.45	2.39	8.78	10.36	17.86	9.40	35.40	12.27	18.72
PD*N*P			6.29	24.97	10.52	14.29	22.86	28.69	35.47	15.28	8.87	4.09
ANOVA Analysis												
PD			***	***	***	***	***	***	***	***	ns	***
N			***	***	***	***	***	***	***	***	*	***
P			***	***	***	***	***	***	***	***	ns	***
PD*N			***	***	***	***	***	***	***	***	ns	***
PD*P			***	***	***	***	***	***	***	***	ns	***
N*P			***	***	***	***	***	***	***	***	ns	***
PD*N*P			***	***	***	***	***	***	***	***	ns	***

Significant differences among the treatments were detected by the Duncan test (P < 0.05). Parameters included EFW, ear fresh weight; CFW, cob fresh weight; EL, ear length; CL, cob length; ED, ear diameter; CD, cob diameter; KRN, kernel row number; KNPR, kernel number per row; GN, grain number; BTL, barren tip length; and CV, coefficient of variation. Values followed by different lowercase letters within a column are significantly different among treatments (*P* < 0.05). *P < 0.05; **P < 0.01, ***P < 0.001, ^ns^no significance.

### 3.12 Relationships between root system architecture, root biomass, and the root lodging rate

**Figure 12:**
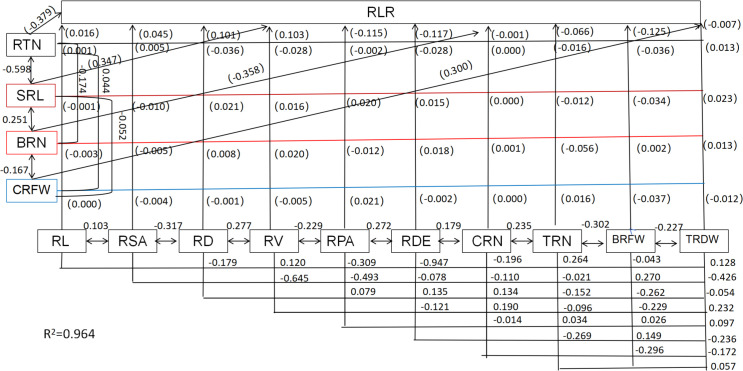
Path analysis model diagram showing causal relationships of root traits and root lodging. Values in parentheses are direct path coefficients, whereas other values are correlation coefficients. RL, Root length; RSA, root surface area; RD, root diameter; RTN, root tips number; RV, root volume; RPA, root projected area; RDE, root density; SRL, specific root length; CRN, crown root number; BRN, brace root number; TRN, total root number; CRFW, crown root fresh weight; BRFW, brace root fresh weight; TRDW, total root dry weight.

Root system architecture is critical for root lodging in sweet corn. Significant negative correlations between root diameter, root surface area, root length, root projection area, number of root tips, root volume, and the RLR rate were recorded (**Table 2**; **Figure 12**). However, the root density and specific root length were significantly positively correlated with the RLR (**Table 2**; **Figure 12**). Moreover, the root number and root weight were significantly negatively correlated with the RLR (**Table 2**; **Figure 12**). Path analysis was performed via a stepwise regression model for the RLR and root agronomic traits. About 96.4% of the total variation (R^2^ = 0.964) in root lodging was attributable to these traits (**Figure 12**). The root tip number and brace root number had the greatest direct contributions to root lodging (-0.379 and –0.358, respectively), whereas the specific root length (0.347) and crown root fresh weight (0.300) had the greatest positive effects on root lodging (**Table 2**; **Figure 12**). The greatest indirect effects on root tip number and brace root number were (–0.2266) and (–0.3032), which contributed to root lodging via specific root length and total root number, respectively (**Table 2**). Moreover, the specific root length and crown root fresh weight had the greatest indirect values of –0.2075 and –0.516, respectively, and contributed to the degree of root lodging through the number of root tips and total root dry weight (**Table 2**; **Figure 12**).

**Table 2:** Direct and indirect effect of root architecture and biomass on sweet corn root lodging.

	RL	RSA	RD	RTN	RV	RPA	RDE	SRL	CRN	BRN	TRN	CRFW	BRFW	TRDW
Coefficient	-0.901	-0.893	-0.912	-0.801	-0.888	-0.908	0.839	0.213	-0.900	-0.790	-0.555	-0.873	-0.847	-0.833
Direct effect	0.016	0.045	0.101	-0.379	-0.103	-0.115	-0.117	0.347	-0.001	-0.358	-0.066	0.300	-0.125	-0.070
Indirect effect														
RL		0.0016	-0.0029	0.0008	0.0019	-0.0049	-0.0152	-0.0006	0.0031	-0.0028	0.0042	0.0000	-0.0007	0.0020
RSA	0.0046		-0.0142	0.0050	-0.0290	-0.0222	-0.0035	-0.0090	-0.0050	-0.0045	-0.0009	-0.0036	0.0121	-0.0197
RD	-0.0181	-0.0320		-0.0357	0.0280	0.0080	0.0136	0.0212	0.0135	0.0080	-0.0153	-0.0057	-0.0264	-0.0054
RTN	0.0193	0.0417	-0.1337		-0.1019	-0.0068	-0.0906	-0.2266	-0.0940	-0.0659	0.0940	0.0167	-0.1076	0.0678
RV	0.0124	-0.0664	0.0285	-0.0277		-0.0234	-0.0125	0.0160	0.0196	0.0202	-0.0099	-0.0049	-0.0234	0.0239
RPA	-0.0355	-0.0567	0.0091	-0.0021	-0.0263		0.03122	0.0197	-0.0016	-0.0116	0.0039	0.0206	0.0030	0.0112
RDE	-0.1108	-0.0091	0.0158	-0.0278	-0.0014	0.0318		0.0154	0.0209	0.0182	-0.0315	-0.0015	0.0174	-0.0276
SRL	-0.0128	-0.0691	0.0729	-0.2075	0.0538	0.0593	0.0458		0.1093	0.0871	-0.0642	-0.0180	0.0937	0.1156
CRN	0.0002	0.0001	0.0001	-0.0002	0.0002	-0.0001	0.0002	0.0003		0.0008	0.0009	-0.0004	0.0002	0.0003
BRN	-0.0637	-0.0354	0.0247	-0.0623	0.0702	-0.0362	0.0558	0.0899	0.2903		-0.3032	-0.0598	0.0057	0.0644
TRN	0.0174	-0.0013	-0.0100	0.0164	-0.0063	0.0022	-0.0178	-0.0122	-0.0561	-0.0559		0.0155	-0.0199	0.0038
CRFW	0.0060	-0.0237	-0.0168	0.0132	-0.0144	0.0537	-0.0039	-0.0156	-0.1131	-0.0501	0.0705		-0.0888	-0.516
BRFW	-0.0054	0.0338	-0.0328	-0.0355	-0.0286	0.0033	0.0186	0.0338	0.0298	0.0020	-0.0378	-0.0370		-0.0284
TRDW	0.0090	-0.0298	-0.0038	0.0125	0.0162	0.0068	-0.165	0.0233	0.0180	0.0126	0.0040	-0.0120	-0.0159	

RL, Root length; RSA, root surface area; RD, root diameter; RTN, root tips number; RV, root volume; RPA, root projected area; RDE, root density; SRL, specific root length; CRN, crown root number; BRN, brace root number; TRN, total root number; CRFW, crown root fresh weight; BRFW, brace root fresh weight; TRDW, total root dry weight.

### 3.13 Relationships between lodging rate, yield, and yield components

**Figure 13:**
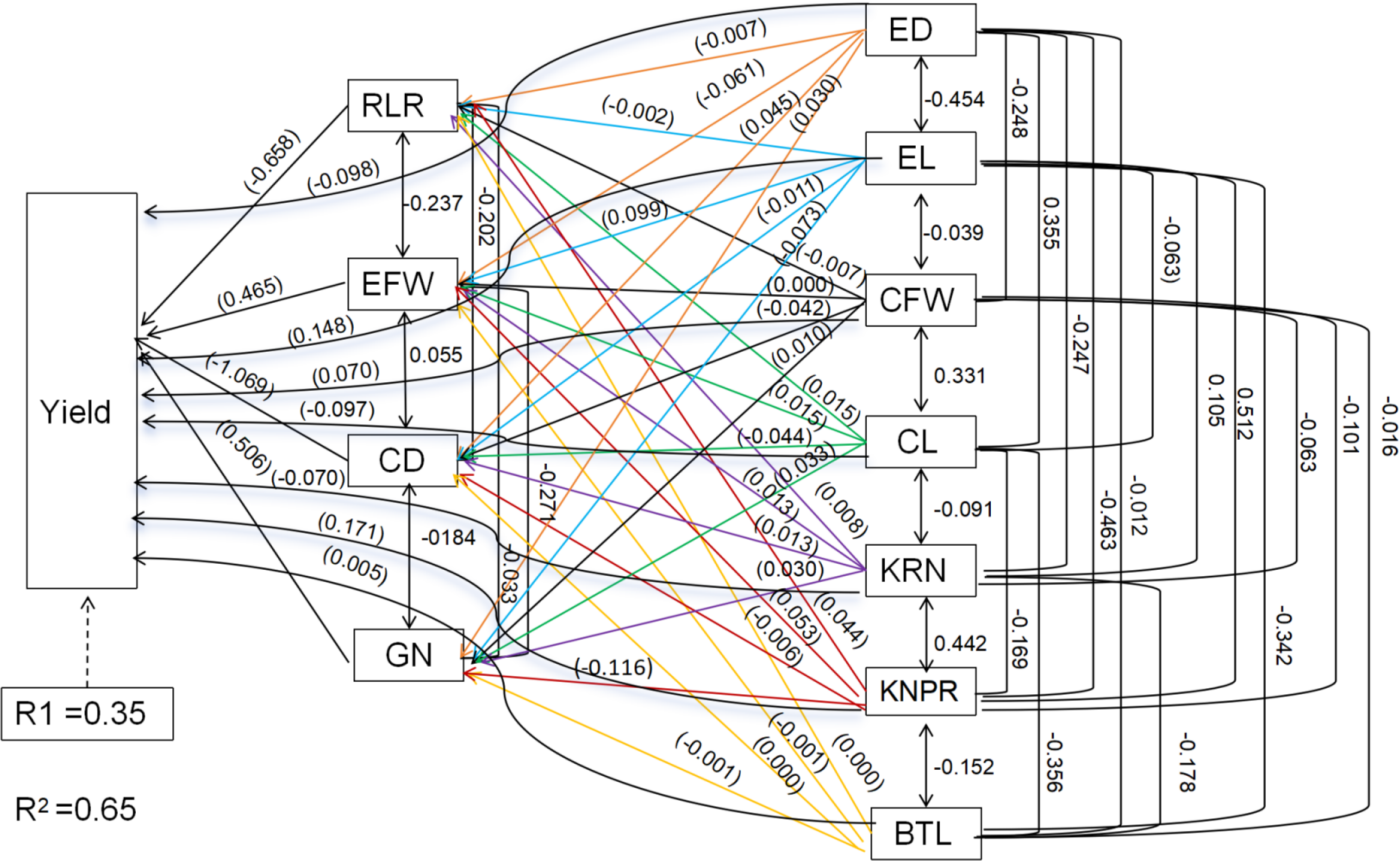
Path analysis model diagram showing causal relationships among ear traits, yield component, root lodging and yield. Values in parentheses are direct path coefficients, whereas other values are correlation coefficients. RLR, lodging rate; EFW, ear fresh weight; ED, ear diameter; EL, ear length; CFW, cob fresh weight; CD, cob diameter; CL, cob length; KNR, kernel row number; KNPR, kernel number per row; GN, grain number; BTL, barren tip length.

Ear traits, yield components and root lodging are crucial factors in determining sweet corn ear yield. **Table 3** shows that ear traits such as ear fresh weight, ear length (0.048), ear diameter (0.069), cob fresh weight (0.007), cob diameter and cob length (–0.404) were extremely weakly related to yield (**Table 3**; **Figure 13**). The yield component kernel row number (0.771), kernel number per row (0.619), and grain number (0.741) were strongly related to yield (**Table 3**; **Figure 13**). The root lodging rate (–0.018) and barren tip length (–0.101) had extremely weak relationships with yield under these field experimental conditions. Path analysis revealed that ear traits, yield components and the root lodging rate accounted for 65.0% (R^2^ = 0.65) of the total variation in ear yield. The results indicated that the root lodging rate had the greatest negative direct effect on ear yield (–0.658), whereas the grain number (0.506) and ear fresh weight (0.465) had the greatest positive direct effects on ear yield. The indirect effect analysis indicated that the root lodging rate had the greatest negative indirect effect (–0.1684) on ear yield via the number of kernels per row. Ear fresh weight (0.3134) and grain number (0.3431) had positive indirect effects on ear yield through ear length and kernel number per row, respectively. (**Table 3**; **Figure 13**).

**Table 3:** Direct and indirect effect of ear traits, yield component and root lodging on ear yield.

	RLR	EFW	ED	EL	CFW	CD	CL	KRN	KNPR	GN	BTL
Coefficient	-0.018	-0.100	0.069	0.048	0.007	-0.103	-0.404	0.771	0.619	0.741	-0.101
Direct effect	-0.658	0.465	-0.098	0.148	0.070	-1.069	-0.097	-0.07	0.171	0.506	0.005
Indirect effect											
RLR		-0.1559	0.0487	-0.0105	-0.0697	-0.1329	0.1013	0.1164	-0.1684	-0.0217	-0.0546
EFW	-0.1102		-0.2902	0.3134	-0.0019	0.0256	-0.0716	0.0842	0.1437	-0.1260	-0.0958-
ED	-0.0072	-0.0612		-0.0445	-0.0243	0.0045	0.0348	-0.0242	-0.0454	0.0296	-0.0012
EL	-0.0024	0.0998	-0.0672		-0.0058	-0.0111	-0.0093	0.0155	0.0758	-0.0728	-0.0506
CFW	-0.0074	-0.0002	-0.0174	-0.0027		-0.0423	0.0232	-0.0044	-0.0071	0.0097	-0.0011
CD	-0.2159	0.0588	0.0492	-0.0802	-0.6467		-0.4864	0.1924	-0.0374	-0.1967	0.1026
CL	0.0149	0.0149	0.0344	-0.0061	0.0321	-0.0441		-0.0088	-0.0164	0.0326	-0.0345
RNPE	0.0082	0.0127	-0.0173	0.0074	-0.0044	0.0126	-0.0064		0.0309	0.0300	-0.0125
KNPR	0.0438	0.0529	-0.0792	0.0876	-0.0173	-0.0060	-0.0289	0.0756		-0.1159	-0.0260
GN	-0.0167	-0.1371	0.1528	-0.2490	0.0698	-0.0931	0.1700	-0.2166	0.3431		0.1361
BTL	-0.0004	-0.0010	0.0000	-0.0017	0.0000	0.0005	-0.0018	-0.0009	-0.0008	-0.0013	

RLR, lodging rate; EFW, ear fresh weight; ED, ear diameter; EL, ear length; CFW, cob fresh weight; CD, cob diameter; CL, cob length; KNR, kernel row number; KNPR, kernel number per row; GN, grain number; BTL, barren tip length.

## 4 Discussion

### 4.1 The metabolism of endogenous phytohormones and root system architecture

Endogenous phytohormones play pivotal roles in regulating crop development and serve as signaling molecules that modulate root system architecture. To date, the underlying mechanism on modulating plant root system architecture by these phytohormones is still unknown. Previous studies have demonstrated that root development is strongly related to auxin, which is a plant hormone that is essential for the formation of primary roots (Yoshida et al., 2013; Pacurar et al., 2014). Compared with other signaling molecules responsible for root development, auxins are considered the most important hormones that guide the root meristem and organogenesis (Bustillo-Avendaño et al., 2018). Additionally, ZR is a vital plant hormone that modulates the formation of primary and lateral roots by stimulating cell division in the root apical meristem (Kuroha and Satoh, 2007). Many studies have revealed that, owing to their synergistic effects on the division and differentiation of root cells, interactions between IAA and ZR in roots induce root formation (Ioio et al., 2008; Kurepa et al., 2018). JA-me may play a role in the negative modulation of primary root length and lateral root length and number by reducing the length of root cortex cells and cell division (Valenzuela et al., 2016). Moreover, other studies have highlighted the synergistic effects of JA-me and IAA on stem cell activation and root regeneration (Marasek-Ciolakowska et al., 2020; Zhang et al., 2023). Several studies have supported the idea that ABA is positively correlated with the root length of plants under environmental stress by stimulating cell division in the meristem and root elongation (Ma et al., 2018). The exogenous application of GA_3,_ which may play a role in the negative regulation of root development, reduces the number of lateral roots and total root length (Wang et al., 2015). During sweet potato storage root formation, the endogenous GA_3_ contents in both adventitious and thick roots are relatively low and have negative effects on root morphology and storage root number (Si et al., 2022). In our study, the changing patterns of IAA, ABA, GA_3_, and JA-me were strongly linearly related to root system architecture and root biomass, which were also significantly positive. In contrast, the ZR content in the roots negatively affected the root system architecture and root biomass. Overall, root system architecture, except for specific root length, and root biomass were negatively correlated with ratios of IAA/ABA, IAA/ZR, IAA/GA_3_, IAA/JA-me, ZR/ABA, ZR/GA_3_, and ZR/JA-me, whereas they were positively correlated with ABA/JA-me, ABA/GA_3_, and GA_3_/JA-me.

### 4.2 Root system architecture and root lodging

The plant root system architecture has pivotal functions in terms of nutrient and water uptake and root anchorage (Rogers and Benfey, 2015). Like in maize, the root system architecture of cereals is composed mainly of crown roots and brace roots (Zheng et al., 2023a). The former refers to the roots that originate at underground stem nodes and are critical for nutrient and water uptake (Zheng et al., 2023a). The latter are known as nodal roots that develop from the stem nodes aboveground and play a role in bracing the plant upright along with nitrogen fixation (Zheng et al., 2023a). Hence, the key challenge is to achieve high yields of sweet corn by coordinating the relationship between the absorption and anchorage functions of the root. The “deep, steep, and cheap” root traits are effective for accessing the resources present in the subsoil and thereby achieving high crop yields (Gao et al., 2022). In summary, an optimal root system architecture is vital for increasing crop development and yield. In the present study, we found significant increases in root length, root diameter, root surface area, root volume, number of root tips, specific root length, and root density with decreasing planting density at both nitrogen application levels during both growth stages. Furthermore, significantly greater values were detected for the plants from the N150 treatment than for those from the N200 treatment during the jointing stage, whereas during the tasseling stage, no significant difference was detected between the different N application rates at a certain planting density, except for the specific root length. The results indicated that in the early stage of crop growth, at reasonably reduced nitrogen rates, the root development of sweet corn is promoted, which can increase morphogenesis in plants. Balancing the relationship between the formed crown roots and brace roots can not only improve the nutrient and water uptake capacity for the development of above-ground plant parts but also strengthen root anchorage and eventually improve lodging resistance in the plant (Rogers and Benfey, 2015). In our study, at a given planting density, the root biomass significantly increased under the N150 treatment because both the root number and fresh weight increased. Notably, the N200 treatment had a comparable effect on maize brace root formation; thus, a greater brace root number was achieved than in N150 plants, whereas the fresh weight of brace roots per plant was lower in these plants than in N150-treated plants. These results demonstrate that the plants under N150 presented greater specific root lengths and crown root numbers, providing more effective access to resources for brace root development. In the present study, the root lodging rate was lowest in D25 plants at both nitrogen application rates but significantly increased with increasing planting density. Here, path analysis revealed that the relationships between the root lodging rate and root system architecture traits as well as the root system biomass were significantly negatively correlated, except for specific root length and root density. These results indicate that root development plays an important role in root lodging resistance. The number of root tips and the number of brace roots had relatively strong direct negative effects on root lodging, whereas the specific length and crown of the roots had positive direct effects on lodging. These findings confirmed that the root absorbing capacity and root anchoring are the main factors affecting the lodging resistance of sweet corn roots.

### 4.3 Lodging-related traits and lodging rate

Previous studies have revealed close relationships among lodging-related characteristics such as plant height, ear height, the coefficient of ear height, the height of the center of gravity, stem node development, dry matter dynamics and plant lodging resistance, with negative correlations with the lodging rate (Liu et al., 2021). The reductions in the plant height, ear height, ear height coefficient, and height of the center of gravity improved the lodging resistance of plants under field conditions. The characteristics associated with stem node development, namely, stem diameter, stem node length, stem-filling traits, and stem node components, play a central role in the modulation of stem lodging (Dong et al., 2023). Overall, a great deal of research has focused mainly on stem lodging. However, little is known about the relationship between the development of stem nodes and root lodging. Lignin, cellulose, and semi-cellulose are the main structural carbohydrates in plant tissues crucial for lodging resistance (Shah et al., 2021; Zhang et al., 2021; Li et al., 2023). In the present study, the decrease in planting density caused significant increases in the plant height, ear height, ear height coefficient, diameter of the 3^rd^ internode, and cross-sectional area of the 3^rd^ internode but significant decreases in the cross-sectional flatness and height of the center of gravity, with average values being lower in N150 plants than in N200 plants. Conversely, significantly greater dry matter accumulation and allocation to both the roots and stems were observed in N150 plants than in N200 plants. Moreover, the root-to-shoot ratio was highest in N150D25-treated plants because nearly greater values of ear leaf photosynthetic traits, such as ear leaf width, length, area, SPAD value, nitrogen content, and angle, were detected in N150D25-treated plants than in their counterparts in the other treatments. Compared with the other treatments, this treatment resulted in greater source capacity and facilitated carbohydrate accumulation and allocation through different plant organs. Hence, the stem lignin content during both growth stages and the root lignin content during the tasseling stage significantly increased in N150D25 plants compared with those in the other treatments and therefore improved the lodging resistance of the plants in this treatment.

### 4.4 Lodging rate, yield and yield components, and yield stability

The traits of commercial ears are important for increasing yield, and the uniformity of ear traits is a crucial factor in determining yield stability (Ali et al., 2017). In our study, in the field experiments, N150 plants presented significantly greater values for the fresh weight and diameter of single ears and cobs, whereas the cob length did not significantly differ among the treatments, and the greatest value of ear length was achieved in N200D25-treated plants. In addition, the N150D25 and N150D20 plants presented the highest yields because of the significant increases in the kernel row number per ear and kernel number per row, respectively. Moreover, the uniformity of ear traits was greater in the N150D25 and N150D30 treatments than in the other treatments in terms of the CV values of the cob fresh weight, ear length, cob length, ear diameter, and kernel number per plant in the former and those of the cob diameter, kernel number per row, grain number, and barren tip length in the latter. We also evaluated the relationship via path analysis. The results indicated that ear traits, e.g., ear fresh weight, ear length, ear diameter, cob fresh weight, cob length, and cob diameter, as well as the root lodging rate and barren tip length, were weakly related to yield, whereas yield components, such as kernel row number per ear, kernel number per row and grain number, showed a significantly positive relation with yield. Among these parameters, the root lodging rate had the greatest negative direct effect on yield, and the grain number and ear fresh weight had greater positive direct effects on yield.

## 5 Conclusion

In the present study, we investigated the metabolism of endogenous phytohormones in roots, lignin biosynthesis, root system architecture, root number, root weight, and stem node and ear leaf development and their relationships with lodging rate, yield, and yield stability at different planting densities and nitrogen application rates. The results proved that reducing the nitrogen rate and increasing the planting density could shape an optimal root system architecture to coordinate the balance between the absorption and anchorage functions of the roots. Hence, improving the development of both the stem node and the ear leaf led to increased dry matter accumulation and allocation to both the root and stem and ultimately improved plant lodging resistance, yield, and yield stability. Our research confirmed that a suitable planting density and nitrogen rate mitigate the lodging risk to plants in the growing season during the summer in tropical regions and that yield loss is caused mainly by root lodging but not stem lodging. However, since root-soil interactions play an important role in plant lodging, studies focused on this topic should consider the soil type. Therefore, further studies are recommended to confirm the findings of the current study.

## Data Availability

The original contributions presented in the study are included in the article/**Supplementary Material**. Further inquiries can be directed to the corresponding authors.
