# Supplementary material for: The agronomic mechanism of root lodging resistance and yield stability for sweet corn in response to planting density and nitrogen rates at different planting dates
Source: Front Plant Sci. 2025 Feb 11;16:1481033. doi: 10.3389/fpls.2025.1481033 (PMC11850264; doi:10.3389/fpls.2025.1481033)
Supplement: Supplementary file 1 [file Table1.docx]

Table S1 Experimental soil physical and chemical properties.

| Growth seasons | Sand content  (%) | Soil density  (g·cm^-2)^ | pH | Organic matter content  (%) | Alkalihydrolysable  nitrogen  (mg·kg^-1^) | Available phosphorus (mg·kg^-1^) | Available potassium (mg·kg^-1^) |
| --- | --- | --- | --- | --- | --- | --- | --- |
| 2023-1 | 49.4 | 1.41 | 6.73 | 1.22 | 58.56 | 29.01 | 73.52 |
| 2023-2 | 48.3 | 1.40 | 6.77 | 1.16 | 63.75 | 28.28 | 66.85 |
| 2023-3 | 48.0 | 1.41 | 6.72 | 1.18 | 62.44 | 36.45 | 78.66 |
