# Supplementary figures and images for: The agronomic mechanism of root lodging resistance and yield stability for sweet corn in response to planting density and nitrogen rates at different planting dates
Source: Front Plant Sci. 2025 Feb 11;16:1481033. doi: 10.3389/fpls.2025.1481033 (PMC11850264; doi:10.3389/fpls.2025.1481033)

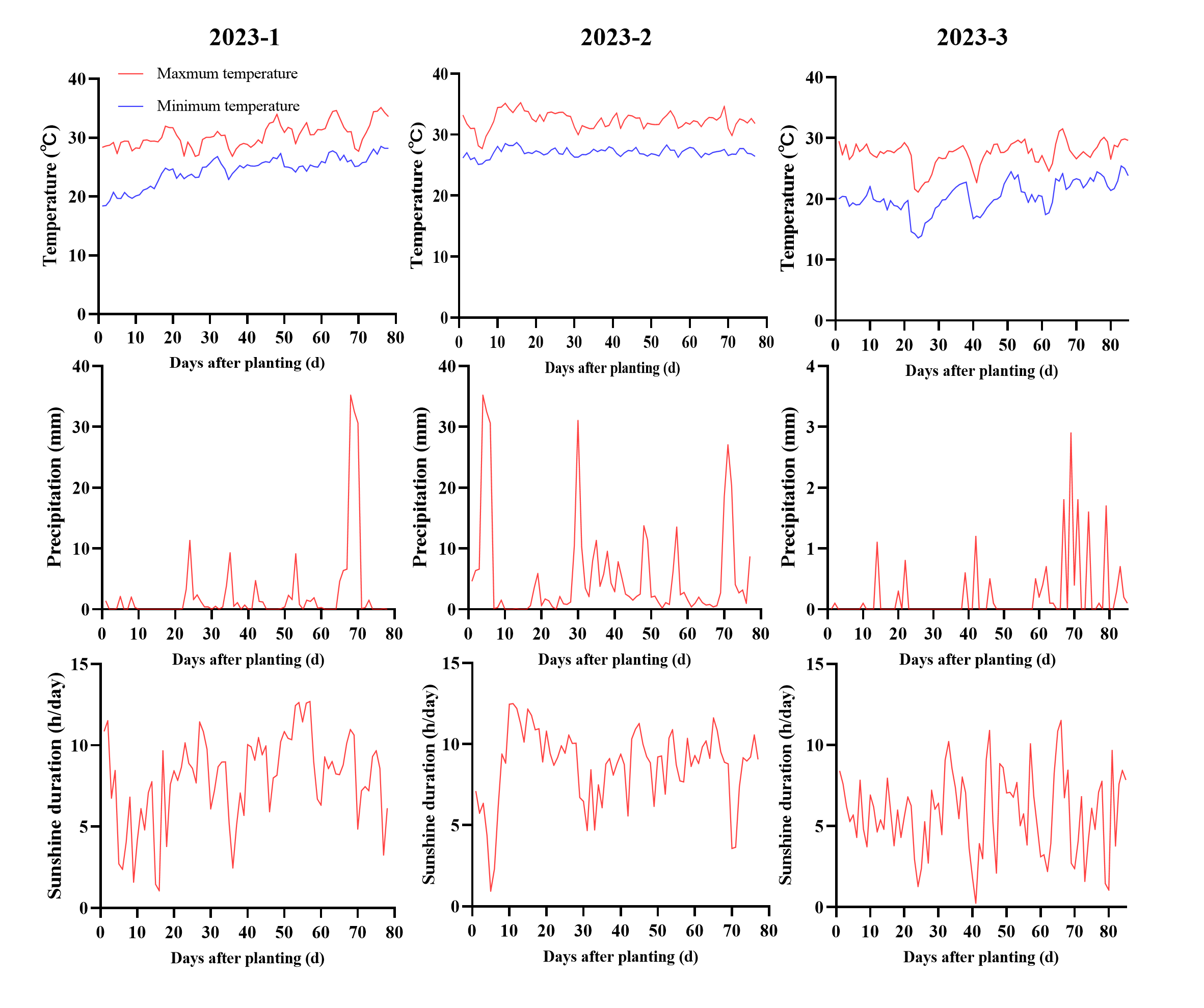

Supplement: Supplementary file 2 [file Image1.tif]
